# Survivability of hospitalized chronic kidney disease (CKD) patients with moderate to severe estimated glomerular filtration rate (eGFR) after experiencing adverse drug reactions (ADRs) in a public healthcare center: a retrospective 3 year study

**DOI:** 10.1186/s40360-018-0243-0

**Published:** 2018-08-29

**Authors:** Monica Danial, Mohamed Azmi Hassali, Loke Meng Ong, Amer Hayat Khan

**Affiliations:** 1 Discipline of Social and Administrative Pharmacy, School of Pharmaceutical Sciences, Universiti Sains Malaysia, 11800 Minden, Penang, Malaysia; 2 Clinical Research Center (CRC) Penang General Hospital, 10990 Jalan Residensi, Pulau Pinang, Malaysia; 3 Discipline of Clinical Pharmacy, School of Pharmaceutical Sciences, Universiti Sains Malaysia, 11800 Minden, Penang, Malaysia

**Keywords:** Chronic kidney disease (CKD), Adverse drug reactions (ADRs), Preventable, Survival rate, Conservative management

## Abstract

**Background:**

Accurate identification and routine preventive practices are crucial steps in lessening the incidence of medications and patients related adverse drug reactions (ADRs).

**Methods:**

Three years retrospective study was conducted among chronic kidney disease (CKD) patients at multi-wards in a tertiary healthcare center. Data collected included demographic characteristics, physical examination results, comorbid conditions, laboratory tests and medications taken. Only medication prescribed during the hospital stay were considered in this study.

**Results:**

From this study only one ADR incident was definitely preventable and majority of other ADRs (88.3%) were possibly preventable. Type of renal replacement therapy (*p =* 0.023) and stages of renal function (*p =* 0.002) were significantly associated with survivability of the hospitalized CKD patients after ADRs. Highest percentage of mortality based on categories were 50–59 years (20.0%), male (16.3%), Indian ethnicity (23.7%), obese (15.0%), smoking (17.1%), consumes alcohol (17.4%), conservative management of renal disease (19.5%) and renal function of < 15 mL/min/1.73m^2^. Overall survivability using Kaplan-Meier analysis reported a significant difference of 18-day survival rate between patients undergoing hemodialysis and patients conservatively managing their renal disease. The 18 days survival rate of patients undergoing hemodialysis, peritoneal dialysis and conservative management were 94.9%, 91.7% and 75.1% respectively. Eighteen days survival rate of patients with renal functions of 30–59 mL/min/1.73m^2^, 15–29 mL/min/1.73m^2^ and < 15 mL/min/1.73m^2^ were 87.4%, 69.8% and 88.6% respectively. Similarly, Cox regression analysis revealed that renal replacement therapy was the only factor significantly contributed to ADRs related mortality. CKD patients whom conservatively managed renal disease or/and with renal function of < 15 mL/min/1.73m^2^ had 5.61 and 5.33 higher mortality risk respectively.

**Conclusion:**

Majority of the reported ADRs were possibly preventable. Renal replacement therapy and/or renal function were significant risk factors for mortality due to ADRs among hospitalized CKD patients stages 3 to 5. Clinician engagement, intensive resources and regular updates aided with online monitoring technology are needed for enhancing care and prevention of ADRs among CKD patients.

## Background

Human body is an intricate system where myriad of biological interactions entangles into a network. Minor disruption in the network by a drug can cause diverse reactions including adverse drug reactions (ADRs). ADRs are caused by the drug interaction with undesired targets within our body [1]. In addition, complex underlying disease states of the human body also influences the drug-drug interaction thus contributing to ADRs. Moreover, factors like increase in the number and type of marketed drugs, increase in aging population, immunological factors (gender and pregnancy), pharmacokinetics differences, polypharmacy and urbanization [1–3] elevates the risk of ADRs. The most commonly reported ADRs causing drugs were NSAIDs, aspirin, anti-neoplastic, anti-psychotics, diuretics and anti-arrhythmic [4]. Tan et al. [1] reviewed, that the top drug-induced toxicities were hepatotoxicity (21%), nephrotoxicity (7%), cardiotoxicity (7%), torsade (21%) and rhabdomyolysis (7%). Each drug prescription carries its own risks for causing ADRs, ranging the full spectrum of severity from cosmetic to severe morbidity and mortality due to patients specific reasons [5].

Clinically significant medications and patients related to ADRs were usually predicted and mostly preventable with few not preventable ADRs [6–8]. Moreover, some of the newly introduced drugs’ side effects were not fully documented hence would possibly exert severe deleterious impact during usage [9]. In recent years, it was reviewed and reported that all drugs cause side effects, however the impact and severity vary and ranges from mild (for example: mild itching or mild headache) to severe (for example: severe rash, damage to vital organs, primarily the liver and kidneys and possibly even death). Therefore, precise diagnosis of ADRs is crucial to reduce preventable ADRs, which however remains a challenge among clinicians [7].

Causality assessment methods are primarily used in evaluating the medication related causality of ADRs [10, 11]. These methods traditionally utilize three approaches such as expert judgement, probabilistic method and algorithm method. More recent approaches are genetic algorithm, Liverpool algorithm and pediatric algorithm [12]. Severity is used for quantification of discomfort grades. Hatwig and colleague [13] developed a scale for assessing the severity of ADRs. Classification on severity are mild (slightly bothersome; relieved with symptomatic treatment), moderate (bothersome, interferes with activities; only partially relieved with symptomatic treatment) and severe (prevents regular activities; not relieved with symptomatic treatment) [14–16]. ADRs preventability are determined by ADRs types which ranges from type A till type D. Type A or Type 1 (augmented) reactions results from an exaggeration of a drug’s normal pharmacological actions when common therapeutic dose administered. Type A is usually dose dependent. Type B or Type 2 (bizarre) are ADRs that occurs as novel response not expected from known pharmacological action [17]. Type C (chronic) ADRs includes adaptive changes, rebound phenomena and other long-term effects. Type D (delayed) reactions are carcinogenesis, affecting reproduction such as impaired fertility and adverse effects on the foetus during early or later stages of pregnancy and drug availability in breast milk [16].

Chronic kidney disease (CKD) is a major health burden that amplifies the risk for adverse events [18, 19]. CKD is independently associated with increased adverse risks including kidney failure, cardiovascular events and all-cause mortality [19, 20]. An eight-year (1999–2006) retrospective study conducted in the United States revealed that there were more than 2 million deaths which were attributed as ADR-related deaths [3]. Additionally, Pirmohamed et al. [21] reported high incidence of in-hospital ADRs which was about 14.7%. Therefore it is beneficial to evaluate patients risk factors for ADRs individually. For minimization of ADRs events, understanding and knowledge on prescribed drug metabolization mechanisms, magnitude and probable ADRs is essentially to be equipped by the healthcare professionals. Thus, these will establish safe medication prescription practices which stresses cautious consideration of the benefits and risks of concomitant medications [22]. Therefore, this study aimed to assess the causality, severity and preventability of ADRs among hospitalized CKD patients with estimated glomerular filtration rate (eGFR) value of < 60 ml/min/1.73m^2^. Additionally, risk factors for mortality due to ADRs were also evaluated.

## Methods

### Study design and participants

This a 3 years retrospective observational study conducted in Penang General Hospital. It is the seconds largest General Hospital in Malaysia. A total of 1070 medical records of patients experienced ADRs from various wards for the duration of 3 years (January 1, 2014 till December 31, 2016) were screened. CKD patients stages 3–5 (eGFR< 60 ml/min/1.73m^2^) with stable serum creatinine (sCr) values during the initial days of admission and experienced ADRs during hospitalization were the primary inclusion criteria of this study. The sCr value obtained during the first day of admission were used to estimate the glomerular filtration rate (GFR). Additional inclusion criteria were patient aged ≥18 years old and admitted for more than 24 h. Medical records which were dubious and incomplete and ward admission due to ADRs or acute kidney injury (AKI) were excluded from this study. Only 160 patients were selected after subsequent screening and identification of records that met the inclusion and exclusion criteria. From the total number of the patient records finally selected, 132 patients survived and 28 patients did not survive ADRs during hospitalization. Prior to study commencement, ethical approval was obtained from Medical Research & Ethics Committee (MREC), Ministry of Health Malaysia (MOH). Study approval number: NMRR-15-1810-28,375(IIR).

### Estimation of renal function

For each patient, the sCr value was measured at admission using the standardized GFR method in the hospital laboratory department. The eGFR was then calculated from serum creatinine value using the chronic kidney disease epidemiology collaboration (CKD-EPI) equation [23]. Stages of CKD included in this study were 3A eGFR 45–59 mL/min/1.73 m^2^, 3B eGFR 30–44 mL/min/1.73 m^2^, 4 eGFR 15–29 mL/min/1.73 m^2^ and 5 eGFR < 15 mL/min/1.73 m^2^. Based on the type of renal replacement therapy, patients with end stage renal disease (ESRD) were divided into hemodialysis, peritoneal dialysis and patients not undergoing any type of dialysis (will be termed as ‘conservative management or conservatively managed renal disease’ in subsequent sections).

### Data collection

For each patient, data was collected retrospectively from the patients’ medical records using a standardized form. Data collected included (a) demographic characteristics such as age and sex; (b) physical examination results such as blood pressure and weight (c) comorbid conditions such as diabetes, hypertension, vascular disease, heart failure, atrial fibrillation and anemia (d) laboratory tests such as serum and biochemical parameters and (e) medications taken before admission, during hospitalization and medications prescribed at discharge. Only medications prescribed during the hospitalization were considered in this study.

### Identifications of ADRs

The primary outcome of this study was to determine the incidence and patterns of ADRs among hospitalized CKD patients stages 3 to 5. Identification of adverse drug reaction (ADR) event was done using a 3-step identification process (trigger list/ physician order, confirmation by an independent reviewer and assessment of causality, severity and preventability of identified ADRs by experienced pharmacist). In this study, ADRs was defined according to Edwards and Aronson [24]. Suspected ADRs were then classified based on the system developed by Rawlin and Thompson [25]. For each suspected ADR, information collected were (a) date start and end of ADR (b) the probable ADRs causative drugs, administered dosage and frequency (c) physical examination and laboratory results (d) reported adverse outcomes such as dizziness and rash. The beginning of the ADR was the date of the clinical or biological diagnosis of the ADR. The end of the ADR was the date of normalization of the effect which was obtained from the ADR reporting form and justified with the date of laboratory examination with normal results or the disappearance of clinical symptoms reported by physicians and pharmacist. If the end date of ADR and the date of the patient demise was reported on the same day therefore the cause of death is regarded as due to ADRs. The ADRs lasted from 1 day to several weeks. Major drug classes that attributed to ADRs were anti-infective, anti-hypertensive, analgesic, statins and anti-diabetic. Furthermore, anti-infectives contributed to highest number of mortality in this study (Danial M, Hassali AMA, Ong LM and Khan AH. Direct cost associated with adverse drug reactions among hospitalized chronic kidney patients in a public healthcare facility: A retrospective 3 year study, submitted).

### Assessment of causality, preventability and severity of ADRs

Assessment of ADRs were done based on causality, severity and preventability. The drug related causality was assessed by using Naranjo algorithm [26]. Only definite and probable ADRs were considered for further assessment. The severity of ADRs were then scored using Hartwig and Siegel [13] scale into mild, moderate or severe. Preventability of ADRs were determined using Hallas et al. [27] criteria into definitely preventable, possibly preventable and non-preventable. The overall incidence of ADRs were defined as the total number of patients who suffered ADRs during hospitalization in relation to the total number of patients admitted to various wards during the 3-year study period.

### Statistical analysis

For the purpose of descriptive analysis, baseline characteristics of patients with ADRs were analyzed using either chi-square test for categorical variables and t-test or Mann-Whitney test, depending on the skewness of data, for continuously distributed variables. The Cox regression analysis was used to estimate of the relative risk of having an ADR during hospital stay in relation to stages of renal function (stages 3 to 5) or for ESRD in relation to the three types of renal replacement therapy (hemodialysis, peritoneal dialysis and conservative management). The Cox regression model is the most frequently used model for analyzing time-to-event data [28]. In this case, the time from hospital admission to day on which the ADR occurred was considered as the time to event and the outcome of the model is either survival or death. The advantage of using Cox regression model is the ability to censor patients who fail to reach the study end-point [29]. In this case, patients who survived of an ADR during the hospitalization were censored. The hazard ratio is the probability that a patient survived the event or the outcome to a certain time point [29]. The hazard ratio survival of the ADR event was reported graphically using the Kaplan-Meier estimates, plotting the log-minus survival function over time. The log-rank test was used to investigate the association with the outcome. All analysis was performed using SPSS (version 22; SPSS Inc., Chicago, IL). Two-sided *p*-values of less than 0.05 were considered statistically significant.

## Results

### Baseline characteristics of patients

Baseline characteristic of patients were similar as reported in (Danial M, Hassali AMA, Ong LM and Khan AH. Direct cost associated with adverse drug reactions among hospitalized chronic kidney patients in a public healthcare facility: A retrospective 3 year study, submitted). CKD patients were grouped into survived (*n* = 132) and whom did not survive after ADRs (*n* = 28) event during hospitalization. Majority of the study patients were Chinese; male; aged ≥60 years; with eGFR value of < 15 mL/min/1.73m^2^ and conservatively managed the renal disease (Table 1). Furthermore, the CKD patients were reported to have comorbidities primarily such as diabetes, dyslipidaemia and hypertension. Additionally, it was reported that they consumed ≥23 of total number of medications (Danial M, Hassali AMA, Ong LM and Khan AH: Development of a mortality score to assess risk of adverse drug reactions among hospitalized patients with moderate to severe chronic kidney disease, submitted).

**Table 1:** Demographic characteristics of the study participants (*n* = 160)

Characteristics	n (%)	Survived (*n* = 132)	Died (*n* = 28)
Demographics			
Age			
≤ 49 years	41 (25.6)	34 (21.3)	7(4.4)
50–59 years	40 (25.0)	32(20.0)	8(5.0)
≥ 60 years	79 (49.4)	66(41.3)	13(8.1)
Gender			
Male	92 (57.5)	77(48.1)	15(9.4)
Female	68 (42.5)	55(34.4)	13(8.1)
Ethnicity			
Malay	52 (32.5)	44(27.5)	8(5.0)
Chinese	68 (42.5)	57(35.6)	11(6.9)
Indian	36 (25.0)	27(18.1)	9(5.6)
Currently or previously smoking	41 (25.6)	34(21.3)	7(4.4)
Currently or previously consumed alcohol	23(14.4)	19(11.9)	4(2.5)
Renal Replacement Therapy			
Haemodialysis	61 (38.1)	52(32.5)	9(5.6)
Peritoneal dialysis	12 (7.5)	10(6.3)	2(1.3)
Conservative management	87 (54.4)	70(43.8)	17(10.6)
Renal Function			
30–59 mL/min/1.73m^2^	49 (30.6)	46(28.7)	3(1.9)
15–29 mL/min/1.73m^2^	29 (18.1)	22(13.8)	7(4.4)
< 15 mL/min/1.73m^2^	82 (51.2)	64(40.0)	18(11.3)

### Causality assessment of ADRs

**Fig. 1:**
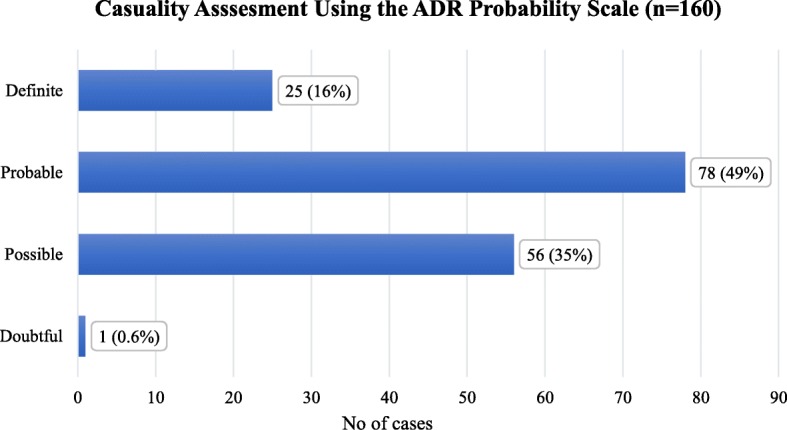
Causality Assessment of CKD patients whom experienced ADR during hospital stay

Based on Naranjo scale there were 25 (15.6%) definite, 78 (48.8%) probable, 56 (35.0%) possible and 1(0.6%) doubtful ADRs respectively (Fig. 1). Cumulatively, the definite and probable ADRs accounted for 103 (64.4%) of the total ADRs. Subsequently, type A accounted for 89 (86.4%) ADRs as per the Rawlin and Thompson classification system. Preventability assessment using Hallas et al. [27] criteria indicated that only one ADR incident was definitely preventable, 91 (88.3%) were possibly preventable and 11 (10.7%) of incidence were non-preventable. Cumulatively, preventable ADRs were about 92 (89.3%) (Table 2). Severity assessment using modified Hartwig and Siegel scale categorized 14 (13.6%) severe, 61 (59.2%) moderate and 28 (27.2%) mild ADRs (Table 3).

**Table 2:** Classification and preventability assessment of the ADRs

Assessments of ADRs	No.*	(%)
Classification of ADR based on;		
^a^Type A	89	86.4
^a^Type B	14	13.6
^b^Definitely preventable	1	1
^b^Possibly preventable	91	88.3
^b^Non-preventable	11	10.7

Abbreviations: ADR, adverse drug reaction

*The total number of definite or probable ADRs, *n* = 103

^a^ Type A and Type B ADR are classified based on Rawlin and Thompson (1991)

^b^ Definitely preventable, Possibly preventable and Non-preventable are classified based on Hallas et al. [27]

**Table 3:** Severity assessment of the ADRs

Level	Description	Scale	No.*	(%)
1	An ADR occurs but requires no change in treatment with the suspected drug.	Mild	7	6.8
2	The ADR requires that the suspected drug be withheld, discontinued or otherwise changed. No changed antidote or other treatment is required, and there is no increase in length of stay.	Mild	21	20.4
3	The ADR requires that the suspected drug be withheld, discontinued or otherwise changed, and/or an antidote or other treatment is required, and there is no increase in length of stay.	Moderate	31	30.1
4	a) Any level 3 ADR that increases length of stay by at least one day, or (b) The ADR is the reason for admission	Moderate	30	29.1
5	Any level 4 ADR that requires intensive medical care.	Severe	13	12.6
6	The adverse reaction causes permanent harm to the patient.	Severe	0	0
7a	The adverse reaction indirectly linked to the death of patient.	Severe	1	1.0
7b	The adverse reaction directly linked to the death of patient.	Severe	0	0

Abbreviations: ADR, adverse drug reaction

*The total number of definite or probable ADRs, *n* = 103

### Logistic regression

Categories that were more prone for mortality after ADR events were patients aged 50–59 years (20.0%); male (16.3%), Indian ethnicity (23.7%); obese (15.0%); with current or past history of smoking (17.1%); with current or past history of alcohol consumption (17.4%); conservatively managed renal disease (19.5%) and with renal function of < 15 mL/min/1.73m^2^ (Table 4). The multiple logistic regression values indicated that the age category of 50–59 years had 2.05 (95% CI 0.57–7.34) higher rates of not surviving ADRs compared to age categories of ≤49 years and ≥ 60 years old. Additionally, males had higher mortality rate than females. Indian ethnicity had 2.59 (95% CI 0.77–8.72) higher death rates compared to Malay and Chinese ethnicity. Lowest mortality rate was reported in Chinese ethnicity (OR: 0.70; 95% CI 0.20–2.44). BMI category obese (OR: 1.34; 95% CI 0.18–9.71) had higher rates of mortality compared to overweight, normal and underweight after ADRs. CKD patients with current or past history of smoking and alcohol consumption had higher death rates with OR: 1.19 (95% CI 0.32–4.47) and OR: 1.06 (95% CI 0.21–5.46) respectively. Furthermore, patients whom conservatively managed their renal disease had higher death rate (OR: 5.90; 95% CI 1.63–21.34) compared to those undergoing peritoneal dialysis and hemodialysis. Similarly, CKD patients with renal function of < 15 mL/min/1.73m^2^ recorded highest mortality rate (OR: 22.37; 95% CI 3.99–125.31) in its category. Overall, types of renal replacement therapy (*p =* 0.023) and renal function (*p =* 0.002) were significant factors that influenced the survivability of the hospitalized CKD patients after ADRs.

**Table 4:** Factors associated with survivability after ADR events using simple logistic and multiple regression

Variables	Survived N(%)	Died N(%)	Crude OR	(95% CI)	*p* -value^a^	Adj. OR	(95% CI)	*p* -value^b^
Age category								
≤ 49 years	34 (82.9%)	7 (17.1%)	1.00(ref.)		0.888	1.00(ref.)		0.490
50–59 years	32 (80.0%)	8 (20.0%)	1.21	(0.40, 3.73)	0.735	2.05	(0.57,7.34)	
≥ 60 years	66 (83.5%)	13 (16.5%)	0.96	(0.35, 2.62)	0.931	1.14	(0.35, 3.66)	
Gender								
Male	77 (83.7%)	15 (16.3%)	1.00(ref.)		0.644	1.00(ref.)		0.829
Female	55 (80.9%)	13 (19.1%)	1.21	(0.54,2.75)		0.88	(0.28,2.75)	
Ethnicity								
Malay	44 (84.6%)	8 (15.4%)	1.00(ref.)		0.544	1.00(ref.)		0.108
Chinese	57 (83.8%)	11 (16.2%)	1.06	(0.39,2.86)	0.906	0.70	(0.20, 2.44)	
Indian	29 (76.3%)	9 (23.7%)	1.71	(0.59,4.93)	0.324	2.59	(0.77,8.72)	
BMI category								
Underweight	14 (82.4%)	3 (17.6%)	1.00(ref.)		0.917	1.00(ref.)		0.825
Normal	58 (84.1%)	11 (15.9%)	0.89	(0.22,3.60)	0.865	0.82	(0.17, 4.05)	
Overweight	43 (79.6%)	11 (20.4%)	1.19	(0.29,4.90)	0.806	1.33	(0.27, 6.56)	
Obese	17 (85.0%)	3 (15.0%)	0.82	(0.14,4.74)	0.828	1.34	(0.18, 9.71)	
Smoking								
No	98 (82.4%)	21 (17.6%)	1.00(ref.)		0.934	1.00(ref.)		0.800
Yes	34 (82.9%)	7 (17.1%)	0.96	(0.38,2.46)		1.19	(0.32, 4.47)	
Alcohol consumption								
No	113 (82.5%)	24 (17.5%)	1.00(ref.)		0.988	1.00(ref.)		0.943
Yes	19 (82.6%)	4 (17.4%)	0.99	(0.31, 3.18)		1.06	(0.21, 5.46)	
Renal replacement therapy								
Hemodialysis	52 (85.2%)	9 (14.8%)	1.00(ref.)		0.751	1.00(ref.)		0.023*
Peritoneal dialysis	10 (83.3%)	2 (16.7%)	1.16	(0.22,6.17)	0.866	1.21	(0.21, 6.940)	
Conservative management	70 (80.5%)	17 (19.5%)	1.40	(0.58,3.40)	0.453	5.90	(1.63,21.34)	
Renal function								
30–59 mL/min/1.73m^2^	46 (93.9%)	3 (6.1%)	1.00(ref.)		0.062	1.00(ref.)		0.002*
15–29 mL/min/1.73m^2^	22 (75.9%)	7 (24.1%)	4.88	(1.15,20.69)	0.032	8.90	(1.76, 44.94)	
< 15 mL/min/1.73m^2^	64 (78.0%)	18 (22.0%)	4.31	(1.20,15.51)	0.025	22.37	(3.99,125.31)	

Note: ^a^Simple Logistic Regression;

^b^Multiple Logistic Regression;

Crude OR = Crude Odds Ratio;

Adj. OR = Adjusted Odds Ratio;

95% CI = 95% confidence interval

*p value< 0.05

### Kaplan-Meier overall survivability

**Fig. 2:**
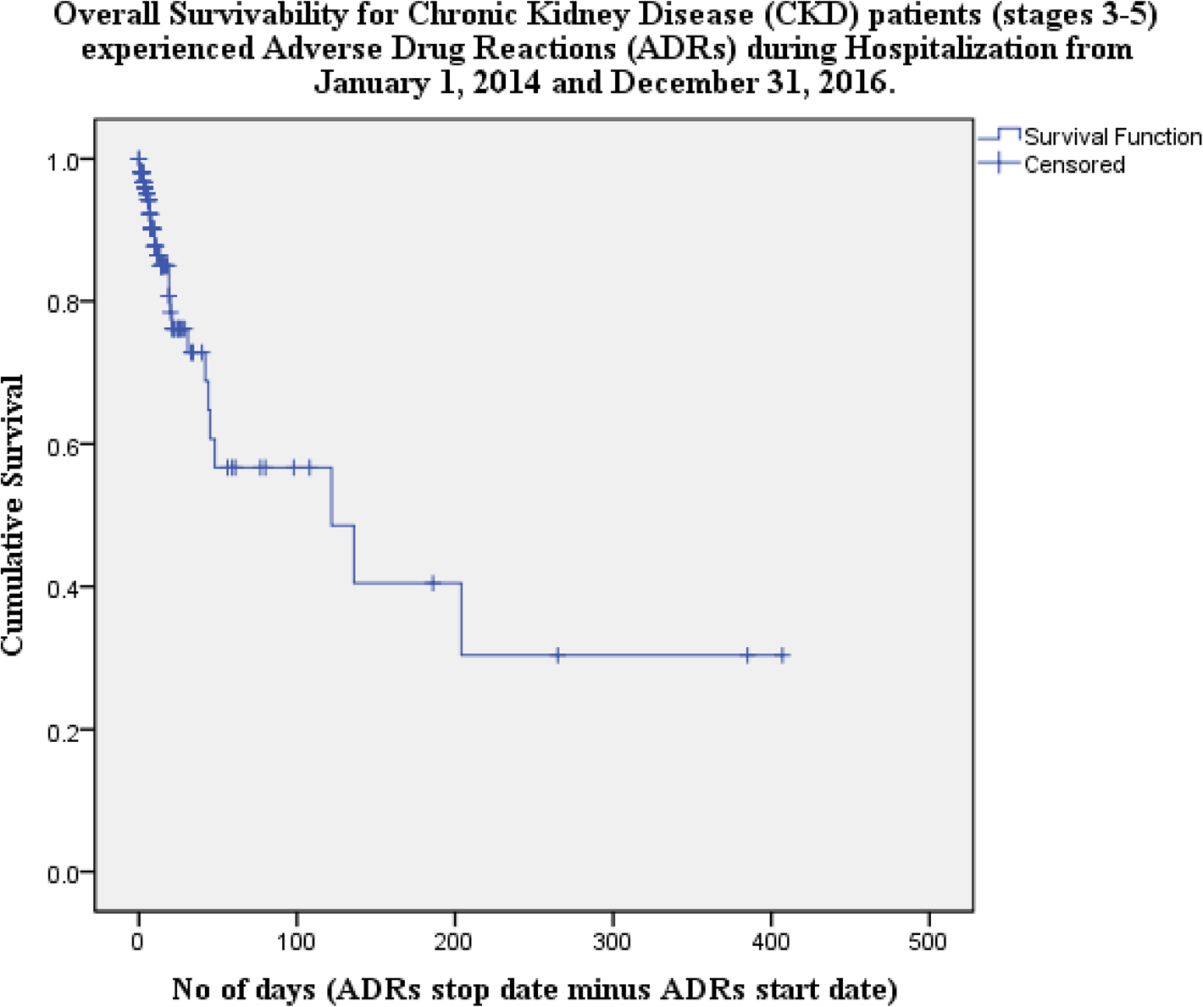
Overall Survivability for Chronic Kidney Disease (CKD) patients (stages 3–5) experienced Adverse Drug Reactions (ADRs) during Hospitalization from January 1, 2014 and December 31, 2016

The Kaplan-Meier overall survivability analysis performed indicated 85.0% survival for the duration of 18 days (Fig. 2). The Kaplan-Meier survival analysis revealed the differences in survival after ADRs among patients with different renal replacement therapy. Eighteen days survival rates of patients undergoing hemodialysis, peritoneal dialysis and conservative management were 94.9%, 91.7% and 75.1% respectively. However, no survival differences were observed in categories of age, gender, ethnicity, BMI, smoking status, alcohol consumption status and renal function. Eighteen days survival rates of age groups ≤49 years, 50–59 years and ≥ 60 years were 86.2%, 85.6% and 84.2% respectively. Eighteen days survival rates based on gender were 84.7% male and 85.5% female. Eighteen days survival rates based on ethnic groups were 86.1% Malay, 80.0% Chinese and 94.7% Indian. Eighteen days survival rate of patients based on BMI categories were 63.0% underweight, 92.0% normal, 73.0% overweight and 93.8% obese. Eighteen days survival rate of patients based smoking status were 84.5% non-smokers and 86.6% smokers. Eighteen days survival rate of patients-based alcohol consumption were 84.5% no and 87.7% yes. Eighteen days survival rate of patients with renal functions of 30–59 mL/min/1.73m^2^, 15–29 mL/min/1.73m^2^ and < 15 mL/min/1.73m^2^ were 87.4%, 69.8% and 88.6% respectively (Table 5).

**Table 5:** Survivability of the CKD patients after ADR event

Variables	Number of Patients	Number of Events	Survivalibility (%)						Comparison	*p* -value^a^
	n	n	3 days	6 days	9 days	12 days	15 days	18 days		
Overall Survivalibility	160	28	96.7	94.2	87.7	86.4	86.4	85.0		
Age category										0.927
≤ 49 years	41	7	95.1	95.1	95.1	91.3	86.2	86.2		
50–59 years	40	8	97.1	97.1	97.1	85.6	85.6	85.6		
≥ 60 years	79	13	97.3	92.5	84.2	84.2	84.2	84.2		
Gender										0.787
Male	92	15	96.6	96.6	91.3	84.7	84.7	84.7		
Female	68	13	96.9	91.1	88.7	88.7	85.5	85.5		
Ethnicity										0.796
Malay	52	8	97.8	97.8	97.8	90.4	86.1	86.1		
Chinese	68	11	96.8	90.6	80.0	80.0	80.0	80.0		
Indian	38	9	94.7	94.7	94.7	94.7	94.7	94.7		
BMI category										0.206
Underweight	17	3	93.3	93.3	84.0	84.0	63.0	63.0		
Normal	69	11	94.0	92.0	92.0	92.0	92.0	92.0		
Overweight	54	11	100.0	94.7	85.3	77.9	73.0	73.0		
Obese	20	3	100.0	100.0	100.0	93.8	93.8	93.8		
Smoking										0.943
No	119	21	97.3	95.2	89.9	86.5	84.5	84.5		
Yes	41	7	95.1	91.2	91.2	86.6	86.6	86.6		
Alcohol consumption										0.647
No	137	24	96.9	94.0	87.7	86.2	84.5	84.5		
Yes	23	4	95.7	95.7	95.7	87.7	87.7	87.7		
Renal replacement therapy										0.050
a. Hemodialysis	61	9	100.0	100.0	97.8	94.9	94.9	94.9	a vs b	0.874
b. Peritoneal dialysis	12	2	91.7	91.7	91.7	91.7	91.7	91.7	a vs c	0.019
c. Conservative management	87	17	95.2	90.1	83.6	78.4	75.1	75.1	b vs c	0.293
Renal function										0.347
30–59 mL/min/1.73m^2^	49	3	100.0	100.0	100.0	87.4	87.4	87.4		
15–29 mL/min/1.73m^2^	29	7	93.0	93.0	82.6	76.7	69.8	69.8		
< 15 mL/min/1.73m^2^	82	18	96.2	92.0	88.6	88.6	88.6	88.6		

Note: ^a^survival analysis using Kaplan-Meier

### Cox regression

The Cox regression analysis revealed that only renal replacement therapy contributed significantly to mortality associated with ADRs among CKD patients. Factors such as age, gender, ethnicity, BMI, smoking status, alcohol consumption status and renal function were not significantly linked to mortality due to ADRs among CKD patients (Table 6). CKD patients whom were conservatively managing their renal disease had 5.61 more risk of dying compared to those whom were undergoing hemodialysis. Additionally, lowest risk of mortality (0.61) were observed in patients undergoing peritoneal dialysis. In terms of renal function, CKD patients with renal function of < 15 mL/min/1.73m^2^ had 5.33 higher risk of mortality compared to patients with renal function of 30–59 mL/min/1.73m^2^ and 2.91 higher risk of mortality compared to patients with renal function of 30–59 mL/min/1.73m^2^.

**Table 6:** Cox regression of the CKD patients after ADR event

Variables	Cencored		Events		Adj. HR	(95% CI)	*p* -value^c^
	n	(%)	*n*	(%)			
Age category							0.934
≤ 49 years	34	82.9	7	25.0	1.00	(ref.)	
50–59 years	32	80.0	8	28.6	1.08	(0.34,3.39)	
≥ 60 years	66	83.5	13	46.4	0.89	(0.31,2.58)	
Gender							0.504
Male	77	83.7	15	53.6	1.00	(ref.)	
Female	55	80.9	13	46.4	0.69	(0.23,2.08)	
Ethnicity							0.997
Malay	44	84.6	8	28.6	1.00	(ref.)	
Chinese	57	83.8	11	39.3	1.02	(0.33,3.11)	
Indian	29	76.3	9	32.1	1.05	(0.33,3.35)	
BMI category							0.434
Underweight	14	82.4	3	10.7	1.00	(ref.)	
Normal	58	84.1	11	39.3	0.94	(0.21,4.25)	
Overweight	43	79.6	11	39.3	1.47	(0.32,6.75)	
Obese	17	85.0	3	10.7	0.42	(0.06,2.83)	
Smoking							0.886
No	98	82.4	21	75.0	1.00	(ref.)	
Yes	34	82.9	7	25.0	0.91	(0.24,3.37)	
Alcohol consumption							0.987
No	113	82.5	24	85.7	1.00	(ref.)	
Yes	19	82.6	4	14.3	1.01	(0.20,5.03)	
Renal replacement therapy							0.003
Hemodialysis	52	85.2	9	32.1	1.00	(ref.)	
Peritoneal dialysis	10	83.3	2	7.1	0.61	(0.11,3.39)	
Conservative management	70	80.5	17	60.7	5.61	(1.94,16.20)	
Renal function							0.089
30–59 mL/min/1.73m^2^	46	93.9	3	10.7	1.00	(ref.)	
15–29 mL/min/1.73m^2^	22	75.9	7	25.0	2.91	(0.65,12.96)	
< 15 mL/min/1.73m^2^	64	82.5	18	64.3	5.33	(1.18,24.07)	

### Hazard ratio

Age group of 50–59 years old had higher risk of dying (HR: 1.08) compared to ≤49 years and ≥ 60 years old. Additionally, male had higher risk of dying (HR: 1.00) compared to females. Indian patients (HR: 1.05) had higher mortality risk compared to Malay and Chinese patients. Overweight patients had higher risk of dying (HR: 1.47) compared to underweight, normal and obese CKD patients. Non-smokers (HR: 1.00) and patients with current or past history alcohol consumption (HR: 1.01) had higher mortality risk after ADRs during hospitalization compared their respective group categories.

## Discussions

Identification of ADRs among CKD patients will be useful in clinical practice as to implement appropriate care aimed at reducing the number of ADRs. Survival estimation studies are vital for the prediction of impending disease burden, redirection in approaches of disease screening and planning of clinical trials both intervention and observational studies, thus paving ways for more successful understanding among public, healthcare providers and policy makers [30].

Based on our study, in Penang General Hospital yearly about 13.5% of hospitalized CKD patients stages 3 to 5 experienced ADRs. Our findings were similar with meta-analysis study conducted by Lazarou et al. [31] where they reported that 10.9% of patients experienced ADRs of all severities as inpatients and another study by Davies et al. [32], which estimated that between 10 and 20% of patients experienced ADRs during hospitalization. Moreover, from our study about 88% of ADRs were possibly preventable. Similar results were reported by Chan et al. [33], where it was reported that 50% of ADR were preventable. Preventable ADRs were commonly associated with prescription of diuretics, antiplatelet, anticoagulant, antidiabetic and NSAIDs drugs to the patients [34, 35].

Severity assessment using the Hartwig and Siegal indicated that nearly 27% of ADRs scored mild and nearly 60% of ADRs scored at level 3 or below on the Hartwig scale. This indicated that remedial action was performed to treat the ADRs as reported in the patients’ medical records. The reported remedial action was either discontinuation of the suspected drug alone and/or treating with the corrective drug. The outcome of the remedial action resulted in additional laboratory investigations, extra procedures, increment in days of hospitalization, admission to intensive care unit and/or death.

Least number of survivors were from age group of ≥60 years (84.2%) using Kaplan-Meier analysis. It has been reported that death is anticipated after an ADR among patients aged more than 55 years [31, 35]. It is attributable to the presence of high levels of albuminuria with impaired level of eGFR among the older adults [30, 36].

In addition, in this study males were more susceptible to mortality due to ADRs compared to female. The Kaplan-Meier survival analysis revealed that the lowest survival experience for male CKD patients to be 84.7%. Inker et al. [30], indicated that the differences between gender may be attributable to faster progression to ESRD in male compared to female, inaccuracies in estimating equations or lower levels of normal GFR in women [37, 38]. For example, Fan et al. [39] reported that the CKD-EPI equation has slight but non-significant overestimation of GFR in women compared with men. Furthermore, gender differences may also due to immunological and hormonal physiology which influences pharmacodynamics and pharmacokinetics responses, particularly in relation to cardiac and psychotropic medications [40].

In this study, significant positive association were found between renal function and survivability after ADR events (*r* = 0.02, *p* < 0.05). Patients whom conservatively managed renal disease (19.5%) and patients with renal function of < 15 mL/min/1.73m^2^ were at the highest risk of mortality. Metabolic changes among advanced CKD patients have been associated with impaired physical function, which includes reduced muscle function, grip strength and cognition [41]. Furthermore, poorer degree of CKD is associated with higher frequency of the frailty syndrome and higher risk of functional decline over time [42–44].

Safe drug dosage is specifically influenced and associated with individual factors like physical parameters including age and weight; presence of comorbidities; physiological status including renal and hepatic function; current medications usage and previously reported history of allergies [45]. Age influences drug pharmacokinetics and pharmacodynamics activities [46]. Older age influences the rapid accumulation of total body fat and inversely reduces lean muscle mass and water volume. Thus, it impairs the dissemination of many drugs for example benzodiazepines, antipsychotics and opioids [47]. Additionally, reduced water availability rises toxicity levels. This condition results in lengthening of drug elimination half-life which in turn causes undesirable drug side effect such as drowsiness, falls and unwanted dosage build-up [47].

Likewise, aging causes impairment of GFR like decrease in renal size, nephron function and assimilations which are accountable for comorbidities such as hypertension, diabetes and heart failure [48]. Therefore, estimation of GFR is vital when up-taking renally eliminated drugs like dabigatran and metformin as safe dosage needed to be prescribed as to lessen the risk of an ADR [48]. It also have been reported that aging causes decline in liver mass and perfusion, which can adversely affect drugs with high hepatic extraction ratio such as diltiazem, opiates and warfarin [49]. These drugs systemic bioavailability surges with higher accumulation in serum coupled with enhanced adverse drug effect [47].

Pharmacovigilance of ADRs includes detection, assessment, understanding and prevention of adverse effects or any other drug-related problem with the aim of enhancing medication safety and patient care [50]. Likewise, E-pharmacovigilance will be a resourceful tool in drug safety for potentially predicting an ADR likelihood by utilizing previously obtained information such as laboratory investigations [51]. Moreover, drug safety can be established by adopting programmed electronic methods which can render information on past errors of medication and/or dosage and potential medication interactions. Beneficial attributes of electronic prescribing methods have been applauded by a recent systematic review where it was reported to reduce medical errors and adverse drug effects [47, 52].

## Study strengths and limitations

The strength of the current study lies in identifying the ADR event using the 3-step identification process. The study population included all ADR events recorded for 3 years continuously from multiple wards, representing all clinical specialties commonly found in most acute hospitals. Furthermore, the age distribution of our study population was comparable to figures for all in-patient admissions from other literatures. Thus, this study produces highly reliable results that represents the real-world practices. However, the study limitations were firstly, it was conducted in one hospital and there is likely to be variation between different hospitals because of differences in the local population characteristics and the specialties within the hospitals. Secondly, since this study was performed in one hospital, it may limit the generalizability of the results. Finally, survival risks for specific stages may be overestimated because it was derived using only one measurement of GFR.

## Conclusions

Conclusively, from this study only one ADR incident was definitely preventable and majority of other ADRs were possibly preventable. Renal replacement therapy and/or renal function were significant risk factors for mortality due to ADRs among hospitalized CKD patients stages 3 to 5. There is a need to develop a high-reliability assessment tool which can meticulously establish suitable diagnostic criteria for ADRs with universal acceptance to improvise the fundamental aspect of drug safety and evades the impending ADRs.

## Abbreviations

ADRs: Adverse drug reactions
CKD: Chronic kidney disease
CKD-EPI: Chronic kidney disease epidemiology collaboration
eGFR: estimated glomerulus filtration
ESRD: end stage renal disease
GFR: Glomerular filtration rate
